# DNA Methylation and the Potential Role of Methyl-Containing Nutrients in Cardiovascular Diseases

**DOI:** 10.1155/2017/1670815

**Published:** 2017-11-16

**Authors:** Gang Liu, Peng Bin, Tianwei Wang, Wenkai Ren, Jin Zhong, Jun Liang, Chien-An Andy Hu, Zhaoying Zeng, Yulong Yin

**Affiliations:** ^1^Key Laboratory of Agro-Ecological Processes in Subtropical Region, Institute of Subtropical Agriculture, Chinese Academy of Sciences, National Engineering Laboratory for Pollution Control and Waste Utilization in Livestock and Poultry Production, Hunan Provincial Engineering Research Center of Healthy Livestock, Hunan Co-Innovation Center of Animal Production Safety, Hunan 410125, China; ^2^University of Chinese Academy of Sciences, Beijing 100049, China; ^3^State Key Laboratory of Microbial Resources, Institute of Microbiology, Chinese Academy of Sciences, Beijing 100101, China; ^4^College of Packaging and Printing Engineering, Tianjin University of Science and Technology, Tianjin 300222, China; ^5^Department of Biochemistry and Molecular Biology, University of New Mexico School of Medicine, MSC08 4670, Fitz 258, Albuquerque, NM 87131, USA; ^6^Animal Nutrition and Human Health Laboratory, School of Life Sciences, Hunan Normal University, Changsha, Hunan 410081, China; ^7^College of Animal Science, South China Agricultural University, Guangzhou 510642, China

## Abstract

Patients suffering from cardiovascular diseases (CVDs) experience a low quality of life and increase pressure on healthcare systems both nationally and globally. DNA methylation, which refers to the pathway by which DNA methyltransferase facilitates the addition of a methyl group to DNA, is of critical importance in this respect primarily because the epigenetic modification is implicated in a range of serious conditions including atherosclerosis, CVDs, and cancer. Research findings indicate that the number of epigenetic alterations can be elicited (both in utero and in adults) through the administration of certain nutritional supplements, including folic acid and methionine; this is partly attributable to the effect employed by methyl-containing nutrients in DNA methylation. Thus, for the purpose of illuminating viable therapeutic measures and preventive strategies for CVDs, research should continue to explore the intricate associations that exist between epigenetic regulation and CVD pathogenesis. This review centers on an exposition of the mechanism by which DNA methylation takes place, the impact it has on a range of conditions, and the potential clinical value of nutrition, driven mainly by the observation that nutritional supplements such as folic acid can affect DNA methylation.

## 1. Introduction

Dietary factors are chief determinants of CVD pathogenesis, and despite the fact that the precise way in which this takes place still remains unclear, Zhu et al. suggest that epigenetic processes may be significant [1]. In view of the centrality of epigenetics to this discussion, it is useful to highlight that epigenetics can generally be described as the modification of gene expression that arises concurrently with no such modification in DNA sequence [2]. Several epigenetic studies have further explained that the term denotes the transmissible modifications that arise in gene expression that is not illuminated by alterations in DNA sequence; rather, the mediation of the modifications takes place by chromatin-based mechanisms [3–5]. Chromatin modifications, miRNA, and DNA methylation are among the currently identified epigenetic procedures, and it is also important to recognize that epigenetic control is a central regulatory system that informs the phenotypic variance observed with respect to cell types in multicellular organisms [6].

Significant research has been conducted on DNA methylation, with key findings showing that epigenetic alterations in DNA play a prominent part in regulating gene expression. Importantly, epigenetic alteration occurs in gametogenesis with respect to the genetic information translated onto genes, and the expression or transferal of this information then takes place in the direction of the inheritor's family. Although the importance of DNA methylation and the part it plays in the pathogenesis of a range of diseases and biological procedures is not fully understood, research findings have implicated it in atherosclerosis, CVDs, imprinting conditions, cancer, and the phenomenon of ageing [2]. Nevertheless, a notable finding was reported by Waterland and Jirtle which highlighted epigenomic marks' responses to environmental variance, as is the case with modifications in nutritional status [7]. Epigenomic marks therefore present opportune diet-based objects on which preventive and therapeutic investigations centering on CVD can be focused on. The reversibility of DNA methylation must also be acknowledged, in addition to the role it plays in CVD pathogenesis and the positive impacts that arise from nutritional supplements. These considerations are further explored in the following section.

## 2. DNA Methylation

DNA methylation can be described as the process by which a methyl group is added covalently to the C5 position of cytosine residues [8]. To further specify the nature of the process, Law et al. showed that DNA methylation takes place in the cytosine-paired-with-guanine (CpG) dinucleotide sequences [8]. Further research revealed that DNA methylation also takes place at cytosine residues which are not placed adjacently to guanine residues [9] and Law et al. indicated that the process is implicated in the standard cell-based manipulation of gene expression [8]. This study also found that the regulation of DNA methylation occurs dynamically [10]. The long-held perspective that DNA methylation serves as an epigenetic mark characterized by stability has been challenged by the recent identification of active DNA demethylation in postmitotic cells as well as dynamic DNA methylation and demethylation *in vivo* [11].

The genome-wide demethylation regarding the parental genomes takes place instantaneously following fertilization and continues throughout the preliminary phases of an embryo's development [12]. Concurrent with this, methylation configurations are reprogrammed and the whole process indicates that a part is being played by available heritable epigenetic memory [12]. This suggest that configurations are transferred over the course of somatic cell division during the lifespan. The observation that susceptibility to external factors that can impact methylation may be a feature of this epigenetic reprogramming process is an important consideration, and it is also worth noting that DNA methylation states do not remain constant throughout a lifespan. Scholarly research has not centered on such modifications with CVDs until relatively recently, with prominent areas of study now focusing on atherosclerosis, hypertension, and diabetes [13]. In addition, the tissue- and cell-specific nature of the epigenetic regulation of gene expression and DNA methylation profiles means that susceptibilities are also tissue- and cell-specific and are also informed by the biological sex and lifespan phase of the organism.

DNA methyltransferases (DNMTs) are responsible for facilitating and regulating DNA methylation, and de novo methylation takes place over the course of embryogenesis as a consequence of the presence of DNMT3a and DNMT3b. In contrast, maintenance methylation takes place over the course of somatic cell division as a consequence of the presence of DNMT1 [14, 15]. Evidence suggests that inducement of epigenetic modifications is facilitated by hydrogen peroxide (H_2_O_2_)—a reactive oxygen species (ROS)—and this involves the increasing tightening of DNMT1's binding to chromatin following hydrogen peroxide treatment. Ultimately, this changes the methylation status of CpG regions [16, 17]. One study demonstrated that mouse DNA that lacks genes to code for methylation enzymes such as DNMTs or methylenetetrahydrofolate reductase (MTHFR) will present with hypomethylation, and it also noted that such mouse DNA displays an increased expression of inflammatory mediators [18]. DNA hypomethylation in mice lacking MTHFR has also been identified as occurring prior to the emergence of aortic fatty streaks [19, 20].

Groups of CpGs, known as “CpG islands,” have been linked to gene promoters, most of which are unmethylated. These CpG islands perform a critical function in the regulation of gene expression, as clearly demonstrated in the context of tissue-specific and developmental-specific configurations [10, 21].

Although CpG islands have been identified as being located in both intragenic and intergenic areas, further study is required to ascertain the functioning of each of these domains. The methylation of DNA at sites inside a promoter region or other sites purposed with regulation is frequently linked with the “silencing” of gene expression, and silencing has a similar capacity to arise when DNA methylation is modified external to a promoter area and not internal to a CpG island [22, 23]. Often referred to as a “CpG island shore,” this occurs in sequences approximately 2 kb upstream [10, 21]. It should also be noted that the potential exists for this to impact the transcriptional activation process, and it is also relevant to emphasize that methylated DNA can counteract the transcriptional activation of chromatic structure, thereby causing it to be in the repressive state [24].

## 3. DNA Methylation and CVDs

Although the pathogenesis of atherosclerosis is known to involve the propagation of vascular smooth muscles cells (SMCs), the gathering of lipids, the progression of connective tissue, the intrusion of inflammatory cells, and the process of calcification [25], available knowledge regarding the methylation of genes implicated in atherosclerosis is not extensive. Research has, however, demonstrated that a range of atheroprotective genes including *ESR1* and *ESR2*, which encode the oestrogen receptors ER*α* and ER*β*, respectively, are repeatedly subject to hypermethylation in both human coronary atherosclerotic tissues and the plaque areas of the ascending aorta. Kim et al. found evidence suggesting that atherosclerosis can be counteracted by the presence of ERs in the coronary arterial wall regarding SMCs and endothelial cells (ECs) [26]. In addition, tissue samples from individuals subjected to coronary artery bypass grafting indicated the presence of *ESR1* hypermethylation in several areas including the aorta, internal mammary artery, and saphenous vein, with higher rates also found to be present in coronary artery atherosclerotic plaques [9].

Evidence linking atherosclerosis with DNA methylation has been found in numerous research studies focusing on both humans and animals [6]. In Lund et al., researchers sought to evaluate DNA methylation in the preliminary phases of atherosclerosis, and it was found that apolipoprotein E-deficient (*ApoE*^−/−^) mice displaying vascular lesions are associated with global DNA hypomethylation in aorta and PBMCs [27, 28]. The researchers also identified modifications in the DNA methylation configuration in each tissue category before vascular damage became discernible. In another study, genomic DNA hypomethylation was observed in highly developed atherosclerotic damage in human patients, atherosclerotic damage in *ApoE*^−/−^ mice, and aortic neointima in balloon-denuded New Zealand white rabbits [29].

It can be concluded from these outcomes that DNA methylation is implicated in the pathogenesis of atherosclerosis. Another group of researchers illuminated methylated CpGs in a differential way, linking them to the occurrence of atherosclerosis and endothelial and smooth muscle operation by formulating a DNA methylation map linked to atherosclerosis in human patients [30]. Zaina et al. identified an increase in atherosclerotic damage relating to DNA methylation, thereby outlining the promise associated with the idea of formulating DNA demethylating measures to facilitate viable clinical outcomes [30]. The Singapore Chinese Health Study also noted that men who have a history of CVD or who are associated with CVD risk factors are characterized by a greater leukocyte global DNA methylation status [31]. In addition, the Samoan Family Study of Overweight and Diabetes found a negative correlation between leukocyte LINE-1 methylation status and plasma LDL in a total sample size of 355 [30]. The study also uncovered a positive correlation between leukocyte LINE-1 methylation status and plasma HDL [32]. The key point to note with regard to these findings is that they reinforce the notion that the global DNA methylation profile of peripheral blood leukocytes has the potential to serve as an appropriate biomarker for heightened risk of CVD.

It is also worthwhile to point out that hyperhomocysteinemia is associated with a reduction in the generation of nitric oxide (NO) and VEGF and is similarly linked to a decrease in global DNA methylation levels and the gene-specific methylation of certain promoters [33, 34]. Research has indicated that DNA methylation is implicated in vascular complications linked to heightened Hcy circulating levels [35]. DNMT1 inhibition, DNA hypomethylation, and chromatin remodeling have been shown to facilitate the inhibition of the expression of certain genes, which further mediate Hcy-induced cyclin A (*CCNA2*) gene silencing and growth inhibition in regard to ECs [36, 37].

## 4. Methyl-Containing Nutrients and Potential Roles in CVDs

Nutritional factors are important for a variety of reasons, most notably because they counteract the adverse impacts that result from disease and impact in utero progression [38–40]. Evidence has been published to suggest that certain nutritional supplements have the capacity to control DNA methylation. For example, research findings indicate that the administration of certain nutritional supplements, including folic acid and methionine, can lower the number of epigenetic alterations (in utero and in adults), and this is attributable to the part played by methyl-containing nutrients in DNA methylation [41, 42]. The primary donor of methyl group is S-adenosylmethionine (SAM), which is produced from methionine, and the operation of DNA methyltransferase facilitates the methylation reaction. “Methyl nutrients” is the term frequently used to denote the nutritional components needed to facilitate methylation reactions, with vitamins and amino acids being the two major categories of such nutrients [34]. Examples of vitamins include vitamin B12, choline, folate, and riboflavin, while examples of the amino acids include methionine, serine, and glycine.

The majority of studies addressing methyl nutrient disequilibrium and DNA methylation have focused on folate status and hyperhomocysteinemia (HHcys). Many of these studies have documented the way in which a lack of folic acid can result in global DNA hypomethylation, the key implication of which is the connection to cancer and CVD risk [43–45]. Research has also noted that folic acid and B vitamins facilitate the development of SAM by providing the one-carbon-metabolism with methionine in a direct manner [46]. Vitamin B substitution therapy involves the usage of folic acid, vitamin B6, and vitamin B12 and is an important process in this context; its chief point of value stems from the way in which it lowers the plasma homocysteine (Hcys) level [47]. The methylation of Hcys to produce methionine is underpinned by the presence of folic acids, and reduced serum levels for folic acid have also been implicated in higher serum levels for Hcys [48]. Supplementing one's diet each day with folic acid and specific types of vitamin B can therefore lower plasma Hcys levels. Prognosis for those suffering from CHD is improved, while CVD risk is decreased for those with no health impairment [49]. Owing to the impact that folate has with respect to the metabolism of nitric oxide, recent studies have drawn connections between it and endothelial dysfunction. In research examining individuals suffering from coronary artery disease (CAD), authors have identified that people homozygous for the MTHFR 677C>T variant were associated with higher vascular 5-methyl-THF concentrations, while no disparities were observed regarding vascular total Hcys concentrations [50, 51]. These findings were linked to endothelial dysfunction and vascular oxidative stress.

Nevertheless, the available knowledge of the impact that the significant consumption of folic acid has with respect to methyl metabolism and DNA methylation remains limited. Only one study in the extant literature presents results showing that leukocyte global DNA methylation was not impacted by 800 g per day folic acid supplementation [52]. In contrast, increased RBC folate status has been linked to a decrease in leukocyte global DNA methylation for postmenopausal females [53]. It is clear that further research is required to identify the impact that the significant consumption of folic acid has on gene-specific DNA methylation processes and DNA methylation processes for certain blood cell categories.

Several researchers have reported that DNA methylation may be linked to vascular issues and a higher risk of CVD in conjunction with higher Hcys circulation levels [54]. Numerous projects, focusing on both animals and humans, have identified a link between HHcys and vascular endothelial dysfunction (VED)—conceptualized here as damaged vascular endothelium-dependent vasodilation. This is significant because it serves as a preliminary marker for vascular disease as a condition which frequently foreshadows cardiovascular damage [55]. VED was linked to patients displaying severe HHcys after an oral methionine load [56] and a study focusing on older females noted reduced global DNA methylation in leukocytes with reduced serum folate concentrations and increased overall Hcys concentrations on a low-folate 7-week-long diet [57]. In a similar study on metabolic balance carried out by Jacob et al., postmenopausal females were administered with a low-folate diet for a period of 41 days, with the results showing a decrease in lymphocyte global DNA methylation and plasma folate and a corresponding increase in Hcys [58].

One critical consideration for the interpretation of human-based research outcomes is the nature of the tissue specificity of the association between circulating overall Hcys concentrations and intracellular SAH concentrations. This stems from the fact that the assessment of DNA methylation frequently takes place with respect to the heterogeneous cell population of peripheral blood mononuclear cells (PBMCs). Heightened plasma and lymphocyte SAH concentrations were connected to lower global DNA methylation with respect to leukocytes and lymphocytes in two studies using participants linked to HHcys [59, 60]. However, other studies have also found no evidence for this connection [61, 62]. Ingrosso et al. demonstrated gene-specific linkages of HHcys in regard to DNA methylation for humans [63]. Individuals suffering from kidney failure, thereby being connected to haemodialysis and HHcys, were associated with global DNA hypomethylation when considered in relation to control subjects. A subgroup of individuals displaying the greatest level of acute HHcys also displayed biallelic expression of the imprinted *H19 gene*, and this was attributed to DNA methylation modifications in leukocytes [63].

More research must be conducted regarding the connection between cysteine and CVDs [64]. More specifically, research has found that the cysteine-CVD risk association can be illuminated by the relationship between circulating overall cysteine concentrations and BMI. This supports the widely accepted fact that obese individuals, or those whose BMI is above the recommended upper limit, are more likely to develop CVDs. In the Hordaland Hcys Study, plasma overall cysteine concentrations and BMI and fat mass at baseline were found to be positively correlated [65]. This research suggests that adiposity, a critical determinant of CVD, could be marked by circulating overall cysteine concentrations [64, 65].

Notably, the rapid accumulation of mass was identified when animals consumed large volumes of cysteine or cysteine-rich protein diets [66, 67]. More specifically, after conducting a case-control study for CVD sufferers and healthy controls, a normally distributed correlation for CVD and plasma overall cysteine concentrations was identified (here, the highest level of risk was linked to plasma concentrations of less than 225 mol/L and greater than 300 mol/L) [64]. CVD sufferers were associated with greater plasma overall cysteine concentrations when compared to healthy controls, but it should be noted that the preponderance of results collected suggest that the degree to which the target association is significant is not manifest [68]. As such, it is possible that the primary relationship may be that displayed with respect to adiposity and plasma overall cysteine concentrations.

## 5. Conclusion

The key preventive role that effective nutrition plays in the context of CVDs has been further emphasized by the wealth of epidemiological and clinical research indicating that properly functioning cardiovascular systems are underpinned by DNA methylation and posttranslational chromatic alterations. DNA methylation facilitates the regulation of gene expression due to the way in which it manipulates the transcription of relevant genes, and the process therefore has the capacity to result in epigenetic alteration. Here, methyl-containing nutrients perform an important function in restricting DNA methylation, and in light of this, subsequent studies should continue to examine the utility of therapeutic measures in mitigating the negative effects arising from DNA methylation.
